# The impact of less severe intimate partner aggression on child conduct problems

**DOI:** 10.1002/jcv2.70024

**Published:** 2025-06-26

**Authors:** Hedwig Eisenbarth, Karina Clavijo Saldias, Paul E. Jose, Johannes A. Karl, Karen E. Waldie

**Affiliations:** ^1^ Victoria University of Wellington Wellington New Zealand; ^2^ Stanford University Stanford California USA; ^3^ University of Auckland Auckland New Zealand

**Keywords:** conduct problems, intimate partner aggression, longitudinal, maternal warmth, SDQ

## Abstract

**Background:**

Significant intimate partner aggression (IPA) has been found to negatively impact outcomes of children, such as increased conduct problems (CP). However, it is unclear if forms of IPA that are less severe (e.g., shoving, pushing or yelling) have no, little, or substantial impact on child CP, which would indicate that the intensity (i.e., dosage) of IPA matters. In addition, it is unknown if the impact of IPA on child CP depends on the reporter (mother vs. partner) and on variables such as maternal depression and parenting.

**Methods:**

We investigated the impact of IPA (both mother‐ and partner‐reported), assessed during pregnancy and 9 months postpartum, on child CP at ages 2, 4.5, and 8 years. We also tested both the potential mediating role of maternal depression and moderating role of maternal warmth, reflecting risk and protective factors, respectively. Using longitudinal data from the Growing Up in New Zealand study, we tested path models with 5298 children.

**Results:**

IPA predicted greater child CP for both mother‐ and partner‐reported IPA, but at different age. Maternal depression partly mediated this effect, which was not moderated by maternal warmth.

**Conclusion:**

These findings underscore the importance of exposure to IPA on child development and provides evidence for that impact on behaviour independent of the effects of maternal depression. Positive parenting like maternal warmth seems not to buffer those negative effects.

## Graphical abstract

Children who were exposed to intimate partner aggression in their early development have a higher chance to show conduct problems later in childhood. This effect is only partially explained by maternal depressive symptoms. In addition, maternal warmth cannot buffer this impact.

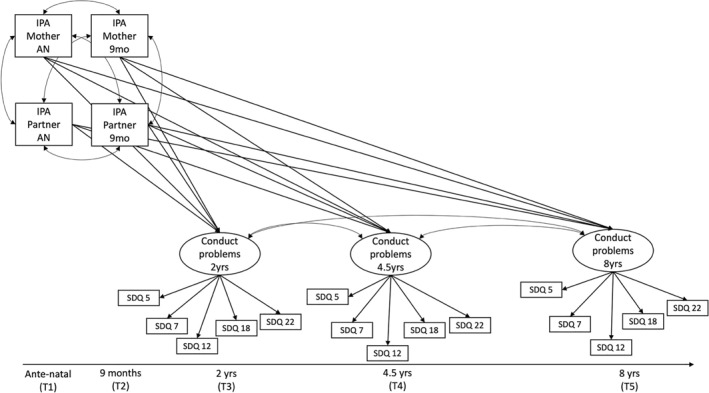

**Key Points**

- Expose to Intimate Partner Aggression (IPA) can have a detrimental effect on children, including long‐term behavioural problems, but unclear if that holds for less severe, more common IPA.
- Mother‐reported IPA had a stronger effect later in childhood, partner‐reported IPA had a more immediate effect on children's behaviour.
- The effect of IPA on children's behaviour problems was partially mediated but not fully explained by maternal depression.
- These findings point out that even less severe IPA experienced in the home needs assessment and intervention for the children in the household.
- Future research needs to replicate the findings and investigate the differential impact of mother and partner reported IPA as well as potential protective factors.

## INTRODUCTION

Growing up in a context of aggressive interpersonal behaviour within the family unit is prevalent and can increase the likelihood for negative child outcomes (Burghart & Backhaus, 2024; Murray & Farrington, 2010). One form of aggressive behaviour that children can be exposed to is intimate partner aggression (IPA) between intimate partners. Central to the *cycle of violence* hypothesis (Abramovaite et al., 2015; Thornberry et al., 2012), children who witness violent behaviour in the household have a higher likelihood of behaving violently later, independent of direct exposure. Impact of lower IPA intensity on children's early conduct, however, is relatively unknown. It is also unclear if it matters who in the intimate relationship is the reporting person for IPA. This study addresses these gaps by investigating the effects of mother‐ and partner‐reported IPA antenatally and in the child's first year on subsequent child behaviour at ages 2, 4.5, and 8 based on the Growing Up in New Zealand longitudinal cohort.

Intimate partner aggression can include various forms. A widely used conceptualisation includes: (i) the intentional use of physical force with the potential for causing death, disability, injury, or harm (physical aggression); (ii) a sexual act that is committed or attempted by another person without freely given consent of the victim or against someone who is unable to consent or refuse (sexual aggression); and (iii) use of verbal and non‐verbal communication with the intent to harm mentally or emotionally, and/or exert control over another person (psychological aggression) (Bagwell‐Gray et al., 2023).

Prevalence estimates of children's exposure to IPA have been of serious concern internationally and in Aotearoa New Zealand (Carlson, 2000; Esquivel‐Santoveña & Dixon, 2012; Fanslow & McIntosh, 2023; Wathen & MacMillan, 2013). Estimating the prevalence of observing aggression in the family, based on rates for lifetime experiences of IPA, most likely underestimates such childhood experiences because such assessments might not include all experiences and because IPA generally is underreported. For instance, though 18.2% of the NZ adult population have reported to have experienced any form of intimate partner violence during their lifetime (NZ Ministry of Justice, 2023), data from the Dunedin Study show that 24% of adults retrospectively reported having witnessed violence or threats of violence directed from one parent to the other before age 18 (Martin et al., 2006). In the US, 39% of children reported witnessing physical IPA, including ‘Grabbing or shoving’ and ‘Kicking, biting, or hitting with a fist’ and 85% reported psychological IPA (Gower et al., 2022) between their parents. These differences might vary based on time of assessment (retrospective as an adult vs. current as a child), reporting person (child vs. parents themselves) as well as types of aggressive behaviour being assessed. Addressing the variability in the assessment of IPA is therefore crucial for a better understanding of the effects of IPA exposure on children.

Across different forms of assessing parental aggression, the negative impact of IPA on children during childhood is documented predominantly for severe forms of IPA. However, IPA manifests on a continuum of severity. According to the most commonly used measure for IPA, the Conflict Tactics Scale (Straus, 2017), ‘severe’ IPA includes kicking, biting, hitting with a fist, hitting or trying to hit with an object, beating up, choking, and burning or scalding, whereas ‘minor’ IPA can include throwing objects, shoving, pushing, grabbing, slapping, and spanking.

The effects of directly observing severe parental physical aggression (direct experience) and of parents reporting IPA in their relationship (indirect experience) on children has been documented, both in retrospective cross‐sectional studies (Martin et al., 2006), cross‐sectional studies in children (Graham‐Bermann & Perkins, 2010), as well as longitudinal studies (Savopoulos et al., 2022). Effects include depression or anxiety in adulthood (Martin et al., 2006), higher externalising and internalising symptoms (Graham‐Bermann & Perkins, 2010), and lower cognitive ability and attention (Savopoulos et al., 2022). Moreover, prenatal stress (e.g., through experiences of IPA of the mother) increases the risk for externalising behaviour in children, even when controlling for post‐natal stressors (Tung et al., 2024). While much of this research has combined ‘severe’ and ‘minor’ forms of physical IPA as dependent variables (Graham‐Bermann & Perkins, 2010; Martin et al., 2006), some studies also report negative effects of less severe forms of IPA (Savopoulos et al., 2022). As less severe IPA is likely more prevalent, understanding effects on behaviour is needed. Moreover, as most research involves maternal reports, partner‐reported IPA could reveal additional information about potential stressors in the child's environment.

### Role of moderators and mediators

Child conduct problems (CP), characterised by aggressive and defiant behaviour, have been identified as a predictor of a wide range of negative outcomes later in life, as well as a marker in the cycle of violence and later anti‐social behaviour (Commisso et al., 2024; Dixon & Graham‐Kevan, 2011; Fong et al., 2019; Moffitt et al., 2001). Risk factors for CP therefore should be considered as potential mediators and moderators between IPA and CP. Maternal mental health is a factor that plays a critical role in developing aggressive behaviour in children (Holmes, 2013). Importantly, experiences of IPA increase mental health problems in parents (Cascardi & O’Leary, 1992). Indeed, the impact of domestic violence on child externalising behaviour at 1 year of age has been found to be partially mediated by maternal mental health in two US based studies (Holmes, 2013; Levendosky et al., 2006). In somewhat older children (grades 1–3) in a Hawaiian sample, a similar mediating effect by maternal depression and parenting stress was found between IPA and externalising behaviours (Bair‐Merritt et al., 2015). In addition, the effects of emotional and physical IPA on cognitive functioning in children have been found to be partially mediated by maternal postpartum depression (Savopoulos et al., 2022). However, these effects have not been tested in the context of a longer developmental timespan or considering different reporting sources for IPA independently.

Another determinative factor for the development of CP is the quality of parenting. Children exposed to both severe IPA and harsh and inconsistent parenting show externalising behaviour, suggesting a buffering effect of effective parental behaviour (Lamela et al., 2018). Similarly, low maternal warmth occurring within the context of severe and less‐severe IPA and maternal depressive symptoms is predictive of mother‐reported higher aggressive behaviour in children (Holmes, 2013). As maternal warmth could buffer the effect of IPA on child outcomes (Holmes, 2013; Skopp et al., 2007), this needs to be considered as a potential moderator for the effects of experiencing IPA on subsequent CP.

Although the effects of experiences of IPA on child aggression in some studies have been found to be independent of age and gender of the child (Gower et al., 2022; Piotrowski et al., 2021), other studies suggest variation depending on basic child characteristics (Fairchild et al., 2019; Skopp et al., 2007). Taken together, the present study addresses the following: Does early exposure to IPA of a less severe type increase child CP? Is this relationship found for both mother‐ and partner‐reported aggression? Does maternal depression mediate the effect of IPA on CP, and can that effect be mitigated by maternal warmth?

## METHOD

### Sample and general procedure

This study is based on the Growing Up in New Zealand longitudinal study sample, which includes more than 6000 children, their mothers/caregivers, and their partners (see Morton et al., 2013). Participants from the Growing Up in New Zealand study (GUiNZ) were recruited from the Auckland, Waikato, and Manukau regions District Health Boards between 2009 and 2010. Pregnant women were informed about the study and given an offer to participate. For those individuals who consented to participate, data were collected over phone interviews, face‐to‐face observations, and surveys across data collection waves, between 2009 and 2022. Mothers' age at birth was *M* = 31.19 (SD = 5.63, range = 15–47). The Growing Up in New Zealand study was approved by the University of Auckland Ethics Committee prior to its commencement.

We included data from the following Data Collection Waves: antenatal phase (time 1); when children were approximately 9 months (time 2); 2 years (time 3); 4.5 years (time 4); and 8 years (time 5). Since the assessment of IPA was crucial, only participants whose mothers were in a relationship at time 1 (antenatal) and time 2 (child 9 months) were included, leading to an analysis sample of *N* = 5298 for the current study (2875 boys, 2423 girls, based on mother‐reported gender of the child at age 4.5, T4). Of those children, mothers reported their ethnicity as predominant Pākeha (*n* = 2798, 52.8%), Māori (*n* = 422, 8.0%), Pasifika (*n* = 505, 9.5%), Asian (*n* = 569, 10.7%), MELAA (Middle Eastern, Latin American and African, *n* = 50, 1.0%) or NZ European (*n* = 500, 9.4%). Ethnicity information was missing for *n* = 454. Reports by the mother during times 2, 3, 4 and 5 was provided predominantly by the biological mother (*n* = 5290), due to the child‐centeredness of the study, the primary caregiver could also be an adoptive mother (*n* = 3), a grandmother (*n* = 2), an aunt (*n* = 1) or another person (*n* = 2). Here we refer to mother informants only as ‘mothers’ for simplicity and because most of these reports did come from mothers. Male partners indicated that they were the biological father (at age 9 months, T2) in 3814 cases (*n* = 2 adoptive father, *n* = 3 stepfather, *n* = 4 other, *n* = 1462 missing). Female partners noted they were the adoptive mother in 3 cases and in another relationship in *n* = 10 cases. Mothers and partners did not change across the time points in our dataset.

### Measures

#### Child conduct problems

We used the Strengths and Difficulties Questionnaire's CP Subscale as reported by the mother (SDQ; Goodman, 1997, 2001). The 5 items include ‘(child) is considerate of other people's feelings', ‘(child) often loses temper’. The mother answered the questions for times 3, 4 and 5 on a three‐point scale with 0 (not true), 1 (sometimes true) or 2 (certainly true) for the 5 statements. The SDQ has previously shown good validity and reliability (Gustafsson et al., 2016; Muris et al., 2003), including for 2‐year‐old children in Aotearoa New Zealand (D’Souza et al., 2017). Higher scores indicate higher levels of CP. One item of the SDQ was missing for all 3801 participants at time 4 (see Table 1 for sum scores and Cronbach's Alpha scores for the current study).

**TABLE 1:** Descriptive statistics and correlation coefficients for all study variables.

Variable	*M*	SD	*r*_*α*_	1	2	3	4	5	6	7	8	9	10
1. IPA mother T1	5.89	2.60	0.70										
2. IPA mother T2	6.21	2.87	0.72	0.61**									
3. IPA partner T1	5.64	2.27	0.66	0.54**	0.46**								
4. IPA partner T2	5.98	2.53	0.70	0.47**	0.58**	0.60**							
5. Conduct T3	2.83	1.73	0.60	0.08**	0.09**	0.13**	0.15**						
6. Conduct T4	1.97	1.58	0.58	0.17**	0.19**	0.11**	0.15**	0.14**					
7. Conduct T5	1.27	1.46	0.61	0.11**	0.14**	0.06**	0.09**	0.10**	0.28**				
8. M Dep T4	3.89	4.07	0.80	0.20**	0.22**	0.13**	0.16**	0.08**	0.21**	0.12**			
9. M Dep T5	4.20	4.49	0.86	0.21**	0.23**	0.14**	0.12**	0.07**	0.17**	0.16**	0.41**		
10. Warmth 4	4.79	0.45	‐	0.01	−0.00	0.01	−0.00	−0.02	−0.06**	−0.05**	−0.03*	−0.01	
11. Warmth 5	4.68	0.61	‐	−0.04**	−0.08**	−0.04**	−0.07**	−0.04**	−0.07**	−0.11**	−0.07**	−0.07**	0.25**

*Note*: *N* = 5298.

Abbreviations: Conduct T3, Conduct Problems at T3 (2 years); Conduct T4, Conduct Problems at T4 (4.5 years); Conduct T5, Conduct Problems at T5 (8 years); IPA MotherT1, mother reported Intimate Partner Aggression at T1 (antenatal); IPA MotherT2, mother reported Intimate Partner Aggression at T2 (9 months); IPA PartnerT1, partner reported Intimate Partner Aggression at T1 (antenatal); IPA PartnerT2, partner reported Intimate Partner Aggression at T2 (9 months); *M*, Mean; M Dep T4, Maternal Depression at T4 (4.5 years); M Dep T5, Maternal Depression at T5 (8 years); *r*_*α*_, Cronbach's alpha; SD, Standard Deviation; Warmth T4, Maternal Depression at T4 (4.5 years); Warmth T5, Maternal Depression at T5 (8 years).

#### Intimate partner aggression

The Interparental Conflict Items were taken from the Stepfamilies and Resilience Study (Pryor, 2004). The four conflict items were ‘During the last 4 weeks, how often did you push and shove each other’, ‘During the past 4 weeks, how often did you throw things at each other when arguing?’, ‘During the past 4 weeks, how often did you yell at each other when angry?’ and ‘During the past 4 weeks, how often did you swear at each other when angry?’. Mothers and partners answered on a 7‐point scale ranging from 1 (*Never*) to 7 (*All the time*). Higher scores indicate a higher frequency of experienced conflict. These conflict items ask about ‘minor’ forms of IPA based on the differentiation between minor and severe items drawn by Straus (2017) in the Revised Conflict Tactic Scales (CTS‐R), which include similar items. No items assessed severe IPA (e.g., punching or hitting with something that could hurt). Sum scores across the four variables were created for each time point, for mothers and partners respectively. Higher scores reflected a higher frequency of IPA experienced (see Table 1 for descriptive statistics and correlations).

#### Maternal depression

We used responses from the Patient Health Questionnaire (PHQ‐9; Kroenke et al., 2001) at times 4 and 5. The PHQ‐9 is a screening tool for presence and severity of depressive symptoms and consists of 9 items. Examples include ‘Over the past 2 weeks, how often have you been bothered by little interest or pleasure in doing things’ and ‘feeling tyred or having little energy’. Mothers/caregivers responded on a 4‐point scale that ranged from 0 (*Not at all*), 1 (*Several days*), 2 (*More than half the days*) and 3 (*Nearly every day*). Sum scores are computed for the nine items. The PHQ‐9 has been found to a valid and reliable screening tool for depression and it has shown an 88% sensitivity for major depressive disorder (Kroenke et al., 2001). The suggested cut‐off score for moderate signs of depression is 10, for moderately severe signs of depression 15, and for severe depression 20 (see Table 1 for descriptive statistics and correlations).

#### Maternal warmth

We used a single item from both times 4 and 5: ‘How often do you do the following things when interacting with (child): I express affection by hugging, kissing, and holding him/her)’. Mothers answered on a 5‐point scale that ranged from 1 (*Never/almost never*), 2 (*Rarely*), 3 (*Occasionally*), 4 (*Often*), and 5 (*Always/almost always*). Higher scores indicated greater displays of Maternal Warmth (see Table 1 for descriptive statistics and correlations).

### Data analysis

In addition to missing data due to items not answered or answered with ‘I don't know’ or ‘I do not want to answer’, item 12 of the SDQ was, in error, not administered at time 4. The Expectation Maximisation (EM) algorithm was used to impute all missing data for all relevant variables (R Package: Amelia, version 1.8.0). EM is an accepted method of data imputation, allowing parameter estimations in probabilistic models and has been found to outperform other commonly used imputation methods such as the standard mean approach and multiple imputation (Ghomrawi et al., 2011; Wirtz et al., 2021).

A multigroup structural equation model (SEM) examined the temporal invariance of the SDQ Conduct Scale after imputing missing data across the three time points to test for suitability of testing growth models (see Supporting Information S1). The models for timepoints 3, 4 and 5 varied significantly, indicating that the latent construct of the SDQ CP subscale varies across time points, therefore path models were selected to test the main hypotheses.

**FIGURE 1:**
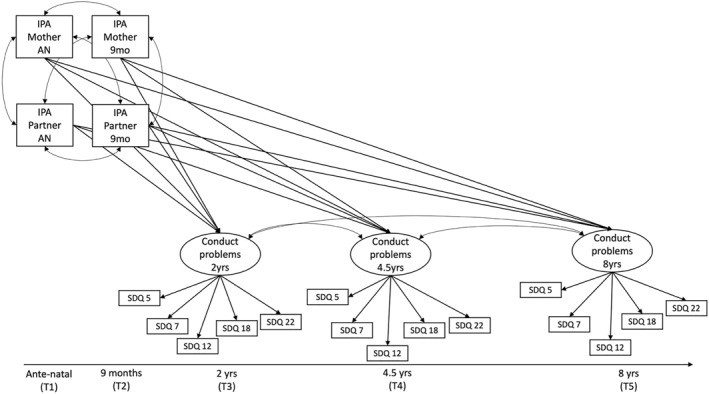
Basic model structure for path analyses. IPA, intimate partner aggression; SDQ, strengths and difficulties questionnaire.

**FIGURE 2:**
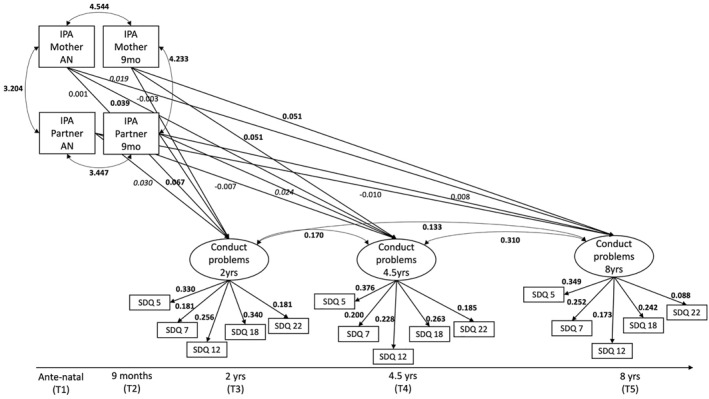
Path model with significant estimates testing the effect of maternal and partner‐reported intimate partner aggression on child conduct problems. IPA, intimate partner aggression; SDQ, strengths and difficulties questionnaire, covariances between each item's timepoints are not shown for the sake of readability, significant estimates are indicated as *p* < 0.001 and *p* < 0.05.

**FIGURE 3:**
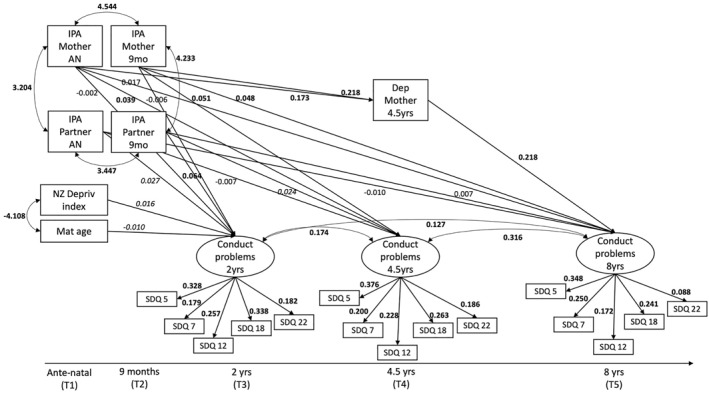
Path model with significant estimates testing the mediating effect of maternal depression for the effect of maternal‐ and partner‐reported intimate partner aggression on child conduct problems. IPA, intimate partner aggression; SDQ, strengths and difficulties questionnaire, covariances between each item's timepoints are not shown for readability, estimates at *p* < 0.001, except when italicised, then *p* < 0.05.

Four structural equation models (SEM) were estimated using the lavaan package in R (Figures 1, 2, 3), and we used the CFI (Comparative Fit Index), Tucker‐Lewis Index (TLI) and RMSEA (Root Mean Square Error of Approximation) model fit indices to interpret the estimated models (Kline, 2023). The criteria for acceptable goodness of fit were: CFI ≥ 0.95, TLI ≥ 0.95 and RMSEA < 0.05 (Schreiber et al., 2006).

Our hypothesised SEM was constructed to investigate the effects of maternal‐ and partner‐reported IPA reported antenatal and at age 9 months on child CP at age 2, 4.5 and 8 years, and it is displayed in Figure 1. Circles represent latent variables and rectangles represent observed variables (see Table 2). Model 2 tested the mediating effect of Maternal Depression at time 4 for the relationship between mother‐reported IPA and CP, controlling for partner‐reported IPA, maternal antenatal age and deprivation (New Zealand deprivation index). Model 3 tested a moderating effect of Maternal Warmth at time 4 for the mediating effect of Maternal Depression of the second model. All reported fit indices are robust, and path estimates in path models are reported in unstandardised format. We chose robust maximum likelihood parameter estimation (MLR) to address that many variables in the models were not normally distributed. We used post‐hoc modification indices to address covariances between indicators of the latent variables (see Figure 2 and S1 for change of model fit indices). Estimates for covariates and latent variables can be found in Tables S3, S4 and S5.

**TABLE 2:** Estimates for regression paths testing the effects of maternal‐ and partner‐reported intimate partner aggression on child conduct problems.

Regression paths	Unstd. estimate	Std. error	*p*	CI lower	CI upper	Std. estimate
IPA M T1						
Conduct T3	0.001	0.009	0.873	−0.017	0.020	0.001
Conduct T4	0.039	0.009	<0.001	0.020	0.057	0.038
Conduct T5	0.019	0.011	0.069	−0.002	0.040	0.019
IPA M T2						
Conduct T3	−0.003	0.009	0.719	−0.020	0.014	−0.003
Conduct T4	0.051	0.009	<0.001	0.034	0.069	0.050
Conduct T5	0.051	0.009	<0.001	0.033	0.070	0.050
IPA P T1						
Conduct T3	0.030	0.010	0.004	0.009	0.050	0.029
Conduct T4	−0.008	0.011	0.492	−0.029	0.014	−0.007
Conduct T5	−0.010	0.011	0.351	−0.032	0.012	−0.010
IPA P T2						
Conduct T3	0.067	0.010	<0.001	0.048	0.087	0.066
Conduct T4	0.024	0.010	0.019	0.004	0.043	0.023
Conduct T5	0.008	0.011	0.435	−0.013	0.029	0.008

*Note*: Underlined *p*‐values differed between full sample versus girls and boys separately (see Supporting Information S1).

Abbreviations: IPA P, Partner reported Intimate Partner Aggression; IPA‐M, mother‐reported intimate partner aggression; Std, Estimates are level standardised; T1, antenatal; T2, 9 months; T3, 2 years; T4, 4.5 years; T5, 8 years.

The hypotheses and data analysis plan were preregistered (https://osf.io/sg9pe/?view_only=8fe69b85a9084fed85e7d580db0cd1c4). All analyses were completed using RStudio software (version 4.0.5, RStudio 2021).

## RESULTS

### Effects of IPA on child conduct problems

We identified significant positive effects of antenatally mother‐reported IPA (T1) on child CP at 4.5 (T4), as well as positive effects of mother‐reported IPA at 9 months (T2) on child CP at 4.5 (T4) and 8 years (T5). We also identified significant positive effects of antenatally partner‐reported IPA (T1) on child CP at 2 years (T3), as well as positive effects of partner‐reported IPA at 9 months (T2) on child CP at 2 (T3) and 4.5 years (T4). The hypothesised model yielded excellent fit to the data (CFI = 0.989; TLI = 0.983; RMSEA = 0.018, 95%CI = [0.015, 0.020]; *Χ* ^2^ (111) = 291.970; *p* < 0.001) (see S1 for model fit indices comparison adding covariances). Using group invariance testing for child gender, only marginal differences in paths were found (see Supporting Information S1).

### Mediation by maternal depression

The hypothesised mediation model was a good fit for the data (CFI = 0.982; TLI = 0.972; RMSEA = 0.022, 95%CI = [0.019,0.024]; *Χ* ^2^ (127) = 397.340; *p* < 0.001). We specified the same covariances between indicators of the latent variables as in the basic model.

Adding the mediating variable Maternal Depression resulted in significant indirect effects of IPA through Maternal Depression on child CP for all mediation paths (see Figure 3). The total effect for Maternal‐reported IPA at 9 months was also significant, while the total effect for antenatal Maternal‐reported IPA was not significant (see Table 3). Direct effects were the same as in the basic model: there were significant direct effects for antenatally mother‐reported IPA on child conduct at 4.5 years, as well as for mother‐reported IPA at 9 months on child CP at 4.5 and 8 years. There were also significant direct effects of antenatally partner‐reported IPA on child CP at 2 years, as well as for partner‐reported IPA at 9 months on child CP at 2 and 4.5 years. Covariances can be found in Supporting Information S1, estimates for latent variables in Supporting Information S1.

**TABLE 3:** Estimates for direct, indirect and total effects for the mediating role of maternal depression for the effect of maternal‐ and partner‐reported intimate partner aggression on child conduct problems.

Regression paths	Unstd. estimate	Std. error	*p*	CI lower	CI upper	Std. estimate
IPA M T1						
Conduct T3	−0.002	0.009	0.807	−0.021	0.016	−0.002
Conduct T4	0.039	0.009	<0.001	0.020	0.057	0.038
Conduct T5	0.017	0.011	0.117	−0.004	0.038	0.017
IPA M T2						
Conduct T3	−0.006	0.009	0.485	−0.024	0.011	−0.006
Conduct T4	0.051	0.009	<0.001	0.034	0.069	0.050
Conduct T5	0.048	0.009	<0.001	0.030	0.067	0.048
IPA P T1						
Conduct T3	0.027	0.010	0.007	0.007	0.048	0.027
Conduct T4	−0.007	0.011	0.493	−0.029	0.014	−0.007
Conduct T5	−0.010	0.011	0.378	−0.032	0.012	−0.010
IPA P T2						
Conduct T3	0.064	0.010	<0.001	0.045	0.084	0.063
Conduct T4	0.024	0.010	0.019	0.004	0.043	0.023
Conduct T5	0.007	0.011	0.482	−0.013	0.028	0.007
Indirect effects						
IPA M T1‐ Mat Dep T4 ‐ conduct T5	0.003	0.001	0.009	0.001	0.004	0.003
IPA M T2‐ Mat Dep T4 ‐ conduct T5	0.003	0.001	0.005	0.001	0.006	0.003
Total effects						
IPA M T1‐ Mat Dep T4 ‐ conduct T5	0.019	0.011	0.070	−0.002	0.040	0.019
IPA M T2‐ Mat Dep T4 ‐ conduct T5	0.052	0.009	<0.001	0.033	0.070	0.051

Abbreviations: IPA P, Partner reported Intimate Partner Aggression; IPA M, mother‐reported intimate partner aggression; Std, Estimates are level standardised; T1, antenatal; T2, 9 months; T3, 2 years; T4, 4.5 years; T5, 8 years.

### Moderating effect of maternal warmth for the mediation by maternal depression

The hypothesised model for the moderating effect of Maternal Warmth for the mediation by Maternal depression effect yielded a good fit for the data (CFI = 0.945; TLI = 0.928; RMSEA = 0.030, 95% CI = [0.028, 0.032]); (*Χ* ^2^ (206) = 1223.279; *p* < 0.001). We used the same covariances between indicators of the latent variables as in the basic model (Table S1).

Direct effects of Maternal Warmth for Conduct both at age 4.5 and 8 were significant and negative. There were two pathways that were not significant that included moderation of the mediating effect of maternal depression: for Maternal antenatal reported IPA on CP at age 8 by Maternal Warmth (Est. = −0.001, *p* = 0.413, 95% CI = [−0.005, 0.002]) and for maternal reported IPA at 9 months on CP at age 8 by Maternal Warmth (Est. = −0.002, *p* = 0.416, CI [−0.006, 0.002]). All indirect effects and the total effect for Maternal‐reported IPA at 9 months were significant, all direct effects for IPA and CP were the same as in the Mediation model (Table S7 and Figure S1).

## DISCUSSION

This study investigated the effects of mother‐ and partner‐reported exposure of their children to physical IPA of less‐severe types during pregnancy and during the first year on children's CP in early (2 years) and mid‐childhood (4.5 and 8 years). In addition, we investigated a potential mediating effect of Maternal Depression potential buffering moderating effect by Maternal Warmth. Path models revealed significant positive effects of mother‐reported IPA on CP in mid‐childhood but not in early childhood, indicating higher IPA predicting more CP. In contrast, a significant positive effect of partner‐reported IPA on CP was found only for early childhood not mid‐childhood, indicating higher IPA predicted more CP. The effects were partially mediated by Maternal Depression but were not buffered by Maternal Warmth.

Our findings show that even less‐severe forms of IPA experienced during pregnancy and in early childhood predict greater CP, which is generally consistent with studies that show such effects for more severe forms of IPA (Graham‐Bermann & Perkins, 2010; Martin et al., 2006; Savopoulos et al., 2022). Interestingly, the associations between IPA and CP varied depending on if reported by the mother or by their partner. While mother‐reported IPA had more distal effects on the child (at age 4.5 and 8), partner‐reported IPA had more proximal effects on the child (age 2), despite both reporting on mutual IPA during pregnancy and the first year of age (9 months), as indicated by high correlations between their IPA reports at both time points (see Table 1). This is a novel finding and needs to be interpreted specifically in the context of IPA as assessed here.

The differences between partner‐reporting may be due to different alignments of the mother's perception of IPA during the antenatal period and in the first year, and the mothers' perception of their child's behaviour. For example, mother's IPA experiences seem not to relate to their perception of higher CP of their child at younger age, but it does when their child is older (4.5 and 8 years). Aggressive behaviour might be seen as more normative at age two, particularly for first time mothers. In contrast, partners' reports of IPA agree with mother's perceptions of the child's behaviour at early age but less so at later age. Also, our effects are small and therefore might vary depending on the reporting person. Mothers tend to be the major caregivers so may more accurately perceive poor behaviour. Our findings need to be replicated in different samples and should include the amount of time partners spend with the child.

It is notable that our question about IPA contained the same wording and importantly did not identify who was perpetrating the IPA. This has several implications. We do not know the direction of the aggression described, which leaves the interpretation of the aggression more open. Respondents could also refer to aggression in the relationship more easily, potentially reducing the under‐reporting bias (Sugarman & Hotaling, 1997; Visschers et al., 2017). As most studies investigating the relationship between IPA and CP have been based on directionally reported IPA, that is, using the (Revised) Conflict Tactics Scale, our results show that a more open responding format still shows similar findings. However, future studies should investigate possible differences between sources and directionality of the aggression.

The effects of IPA on CP did not substantially vary between boys and girls, with only two additional direct effects significant if the two groups were considered separately. This result is in line with several studies showing that associations between predictors and outcome variables are similar for boys and girls, even if the mean level of those variables might vary (Colins et al., 2018; Holmes, 2013; Liu & Vazsonyi, 2024; Milledge et al., 2019). The mechanisms by which IPA impacts CP might be generally similar between boys and girls. However, gender differences in the associations between IPA and CP are heterogeneous and need to be continued to be investigated (Fong et al., 2019).

Given the impact that maternal depression can have on child outcomes and the effect IPA has on maternal internalising, we expected maternal depression to partially mediate the effect of IPA on CP. Our findings supported these hypotheses, consistent with previous research (Hails et al., 2018). Our results further revealed not only an indirect effect but also a significant direct effect of IPA on CP not explained by maternal depression. This new finding highlights the extent to which even less‐severe forms of IPA experienced in early childhood can have significant negative effects on behaviour in early‐ and mid‐childhood which cannot be explained by maternal mental health. This provides further evidence for the effect of exposure to IPA even prenatally and in early childhood increasing externalising behaviours in childhood of children in this large longitudinal sample in Aotearoa New Zealand. These results suggest that it would be valuable to examine mediational pathways through maternal stress (Tung et al., 2024) as well as a reduced ability of caregivers to parent under stress (Levendosky & Graham‐Bermann, 2001). Other potential mechanisms that could explain these effects include impacts of IPA exposure for child cognition (Fong et al., 2019), which have been found to be linked with CP in our sample (Neumann et al., 2024). Implications of these findings point towards a need for early interventions to reduce family stressors and support mothers during pregnancy.

Mothers' depressed affect was assessed here, but the research design did not include partners' depressive symptoms which should be addressed in future investigations. Based on family stress theory (Cox & Paley, 2003), both partners' wellbeing contributes to the family environment and therefore to the emergence of CP. In support, a recent study found that only maternal and not paternal depressive symptoms were related to adolescent delinquency (Liu & Vazsonyi, 2024). Given that IPA could be considered as a form of early life trauma, effects of paternal versus maternal on externalising and internalising symptoms should also be investigated (see e.g. Jongenelen et al., 2023; O’Neill‐Murchison, 2021).

Unexpectedly, maternal warmth exerted no buffering effect on the associations of maternal depression and IPA on CP. Although effective parenting has been found to alleviate some negative effects from IPA or maternal depression (Lamela et al., 2018), here we did not find that maternal warmth reduced the effect of maternal depression or IPA. However, our measure for maternal warmth was an item that reflects physical affection, which could be considered as only one aspect of maternal warmth, not including other aspects such as supportiveness, statements of affirmation, empathy, or appreciation (Cheung & Delany, 2022). In addition, maternal warmth seems to have differential effects on toddlers and children, depending on contingency and context (Lohaus et al., 2001), which could therefore influence the direction and strengths of effects in the context of parenting difficulties due to IPA. Thus, in the context of experiences of IPA and Maternal depression, the potential protective parenting variable of specifically physical maternal warmth does not reduce child behaviour problems. As such, existing systemic interventions addressing parenting in the context of child behaviour should consider the role of experiences of IPA.

### Strengths and limitations

One core strength of this study is the type and size of the sample. The Growing Up in New Zealand study includes children from various backgrounds and has a very high retention rate (96%, Morton et al., 2020), which makes it a high‐quality longitudinal community‐based sample. Another strength is that the sample allowed the use of SEM modelling with latent variables over multiple times of measurement, allowing for the variation in the SDQ CP scale across the early development time points.

This study also has several methodological limitations that are rooted in the assessments of the constructs within this ongoing study and missingness of data. While it can be a strength to ask for experiences of IPA in a non‐directional way, the downside is that directionality of the aggressive behaviour cannot be ascertained. In addition, both predictor‐ and outcome‐variables are mainly assessed through mother reports, which is important to remember when interpreting the associations between those variables. Third party information about child behaviour is unfortunately not available in this dataset. Lastly, maternal warmth was assessed only with a single item, as this was what was available. While this single item might reflect maternal warmth through affection shown towards the child, it is a narrow reflection of maternal warmth.

## CONCLUSIONS

Intimate partner aggression like being pushed or shoved during pregnancy and in the first year of a child has a small but significant impact on child behaviour in early and middle childhood. Children of mothers and partners with such experiences evidence more CP at ages 2, 4.5, and 8 and these effects go beyond the effects of maternal depressive symptoms related to such experiences. These findings in a large representative sample in Aotearoa New Zealand suggest that early interventions that address intimate partner problems in pregnant and young mothers seem to be advised given these results.

## AUTHOR CONTRIBUTIONS

**Hedwig Eisenbarth**: Conceptualization; formal analysis; project administration; supervision; validation; writing—original draft; writing—review and editing. **Karina Clavijo Saldias**: Formal analysis; writing—review and editing. **Paul E. Jose**: Methodology; supervision; writing—review and editing. **Johannes A. Karl**: Formal analysis; methodology; writing—review and editing. **Karen E. Waldie**: Data curation; funding acquisition; methodology; supervision; writing—review and editing.

## CONFLICT OF INTEREST STATEMENT

The authors declare no conflicts of interest.

## ETHICAL CONSIDERATIONS

The Growing Up in New Zealand study was approved by the University of Auckland Ethics Committee and all participants provided informed consent.

## Supporting information

Supporting Information S1

## Data Availability

The data are not publicly available due to privacy or ethical restrictions. Access to data of the Growing up in New Zealand study can be requested from the study website.
