# Supplementary material for: The impact of less severe intimate partner aggression on child conduct problems
Source: JCPP Adv. 2025 Jun 26;6(1):e70024. doi: 10.1002/jcv2.70024 (PMC12973122; doi:10.1002/jcv2.70024)
Supplement: Supplementary file 1 — Supporting Information S1 [file JCV2-6-e70024-s001.docx]

**Supporting Information**

**S0 Results for gender invariance testing**

To test for gender differences, we tested the path model for multigroup (gender) invariance. The model yielded a good fit when including gender as a group variable (CFI = .988; TLI = .982; RMSEA = .018, 95%CI = [.016, .021]); *Χ^2^* (234) = 440.354 (*p* <.001). This result indicates that the model fits both groups well. Only marginal differences were obtained for the direct paths: for the model without group considered, there was no significant effect of antenatal maternal-reported IPA (T1) on Conduct Problems at age 8 (T5) and no significant effect of antenatal partner-reported IPA (T1) on Conduct Problems at age 2 (T3). Both of these effects were significant (*p* =.049) for girls and boys when considering the groups separately. This indicates that considering boys and girls within their respective groups increases the association between IPA and conduct problem variables.

**Table S1**

*Means, Standard Deviations, Skewness, Kurtosis and Standard Error for all model variables*

|  | M | SD | Skew | Kurtosis | SE |
| --- | --- | --- | --- | --- | --- |
| SDQ5_3 | 0.93 | 0.64 | 0.03 | -0.38 | 0.01 |
| SDQ7_3 | 0.7 | 0.54 | -0.06 | -0.42 | 0.01 |
| SDQ12_3 | 0.34 | 0.52 | 1.06 | 0.77 | 0.01 |
| SDQ18_3 | 0.51 | 0.58 | 0.56 | -0.24 | 0.01 |
| SDQ22_3 | 0.34 | 0.49 | 1.07 | 0.72 | 0.01 |
| SDQ5_4 | 0.73 | 0.67 | 0.35 | -0.77 | 0.01 |
| SDQ7_4 | 0.54 | 0.55 | 0.3 | -0.9 | 0.01 |
| SDQ12_4 | 0.22 | 0.44 | 0.09 | 0.06 | 0.01 |
| SDQ18_4 | 0.33 | 0.51 | 1.1 | 0.26 | 0.01 |
| SDQ22_4 | 0.15 | 0.4 | 2.63 | 7.13 | 0.01 |
| SDQ5_5 | 0.56 | 0.64 | 0.47 | -0.41 | 0.01 |
| SDQ7_5 | 0.37 | 0.53 | 0.7 | -0.17 | 0.01 |
| SDQ12_5 | 0.09 | 0.36 | 1.82 | 6.25 | 0.00 |
| SDQ18_5 | 0.21 | 0.46 | 1.14 | 1.56 | 0.01 |
| SDQ22_5 | 0.03 | 0.27 | 1.85 | 10.28 | 0.00 |
| IPA_MotherT1 | 5.89 | 2.6 | 2.35 | 8.29 | 0.04 |
| IPA_MotherT2 | 6.21 | 2.87 | 2.16 | 6.54 | 0.04 |
| IPA_PartnerT1 | 5.64 | 2.27 | 1.56 | 4.06 | 0.03 |
| IPA_PartnerT2 | 5.98 | 2.53 | 1.92 | 7.96 | 0.03 |
| Mdep_T4 | 3.89 | 4.07 | 1.73 | 5.08 | 0.06 |
| Mdep_T5 | 4.2 | 4.49 | 1.27 | 2.76 | 0.06 |
| Warmth_4 | 4.79 | 0.45 | -1.97 | 4.73 | 0.01 |
| Warmth_5 | 4.68 | 0.61 | -1.85 | 5.91 | 0.01 |
| Warmth_4 | 4.79 | 0.45 | -1.97 | 4.73 | 0.01 |
| Warmth_5 | 4.68 | 0.61 | -1.85 | 5.91 | 0.01 |

**Table S2**

*Basic model: Changes in Model fit by adding covariances for the latent variables*

| Laten variables covariances | CFI | TLI | RMSEA | CI |
| --- | --- | --- | --- | --- |
| None | 0.937 | 0.920 | 0.038 | 0.036,0.040 |
| + between same items across timepoints^a^ | 0.962 | 0.945 | 0.032 | 0.029,0.034 |
| + modification indices suggested^b^ | 0.998 | 0.983 | 0.018 | 0.015,0.020 |

*Note*: fit indices are robust; a = SDQ5_3 ~~ SDQ5_4, SDQ5_4 ~~ SDQ5_5, SDQ5_3 ~~ SDQ5_5, SDQ7_3 ~~ SDQ7_4, SDQ7_4 ~~ SDQ7_5, SDQ7_3 ~~ SDQ7_5, SDQ12_3 ~~ SDQ12_4, SDQ12_4 ~~ SDQ12_5, SDQ12_3 ~~ SDQ12_5, SDQ18_3 ~~ SDQ18_4, SDQ18_4 ~~ SDQ18_5, SDQ18_3 ~~ SDQ18_5, SDQ22_3 ~~ SDQ22_4, SDQ22_4 ~~ SDQ22_5, SDQ22_3 ~~ SDQ22_5;

b = SDQ5_5 ~~ SDQ7_5, SDQ12_3 ~~ SDQ22_3, SDQ7_5 ~~ SDQ22_5, SDQ5_4 ~~ SDQ22_4, SDQ12_5 ~~ SDQ22_5, SDQ18_5 ~~ SDQ22_5, SDQ7_4 ~~ SDQ22_4, SDQ5_5 ~~ SDQ22_5, SDQ5_4 ~~ SDQ18_4

**Table S3**

*Basic model: Testing Effect of Maternal and Partner-reported Intimate Partner Aggression on Child Conduct Problems - Covariances*

|  |  | Unstd. Estimate | Std. Error | *p*(>\|z\|) | CI lower | CI upper | Std. Estimate |
| --- | --- | --- | --- | --- | --- | --- | --- |
| IPA_MotherT1 ~~ | IPA_MotherT2 | 4.544 | 0.217 | <.001 | 4.119 | 4.969 | 4.544 |
| IPA_PartnerT1 ~~ | IPA_PartnerT2 | 3.447 | 0.147 | <.001 | 3.158 | 3.736 | 3.447 |
| IPA_MotherT1 ~~ | IPA_PartnerT1 | 3.204 | 0.167 | <.001 | 2.877 | 3.531 | 3.204 |
| IPA_MotherT2 ~~ | IPA_PartnerT2 | 4.233 | 0.212 | <.001 | 3.817 | 4.649 | 4.233 |
| IPA_MotherT1 ~~ | IPA_PartnerT2 | 3.113 | 0.175 | <.001 | 2.77 | 3.457 | 3.113 |
| IPA_MotherT2 ~~ | IPA_PartnerT1 | 2.964 | 0.138 | <.001 | 2.693 | 3.235 | 2.964 |
| SDQ5_3 ~~ | SDQ5_4 | 0.021 | 0.005 | <.001 | 0.011 | 0.031 | 0.021 |
| SDQ5_4 ~~ | SDQ5_5 | 0.066 | 0.005 | <.001 | 0.055 | 0.076 | 0.066 |
| SDQ5_3 ~~ | SDQ5_5 | 0.009 | 0.005 | 0.073 | -0.001 | 0.018 | 0.009 |
| SDQ7_3 ~~ | SDQ7_4 | 0.013 | 0.004 | <.001 | 0.006 | 0.021 | 0.013 |
| SDQ7_4 ~~ | SDQ7_5 | 0.033 | 0.004 | <.001 | 0.026 | 0.04 | 0.033 |
| SDQ7_3 ~~ | SDQ7_5 | 0.007 | 0.003 | 0.032 | 0.001 | 0.014 | 0.007 |
| SDQ12_3 ~~ | SDQ12_4 | <.001 | 0.003 | 0.961 | -0.005 | 0.005 | <.001 |
| SDQ12_4 ~~ | SDQ12_5 | -0.001 | 0.002 | 0.714 | -0.004 | 0.003 | -0.001 |
| SDQ12_3 ~~ | SDQ12_5 | 0.005 | 0.002 | 0.033 | <.001 | 0.009 | 0.005 |
| SDQ18_3 ~~ | SDQ18_4 | -0.005 | 0.003 | 0.149 | -0.011 | 0.002 | -0.005 |
| SDQ18_4 ~~ | SDQ18_5 | 0.015 | 0.003 | <.001 | 0.009 | 0.021 | 0.015 |
| SDQ18_3 ~~ | SDQ18_5 | -0.005 | 0.003 | 0.137 | -0.011 | 0.001 | -0.005 |
| SDQ22_3 ~~ | SDQ22_4 | 0.001 | 0.002 | 0.733 | -0.004 | 0.006 | 0.001 |
| SDQ22_4 ~~ | SDQ22_5 | 0.004 | 0.002 | 0.059 | <0.001 | 0.007 | 0.004 |
| SDQ22_3 ~~ | SDQ22_5 | -0.001 | 0.002 | 0.448 | -0.004 | 0.002 | -0.001 |
| SDQ5_5 ~~ | SDQ7_5 | 0.025 | 0.006 | <.001 | 0.013 | 0.036 | 0.025 |
|  |  | Unstd. Estimate | Std. Error | *p*(>\|z\|) | CI lower | CI upper | Std. Estimate |
| SDQ12_3 ~~ | SDQ22_3 | 0.033 | 0.004 | <.001 | 0.025 | 0.041 | 0.033 |
| SDQ7_5 ~~ | SDQ22_5 | -0.008 | 0.004 | 0.038 | -0.016 | <0.001 | -0.008 |
| SDQ5_4 ~~ | SDQ22_4 | -0.043 | 0.005 | <.001 | -0.052 | -0.034 | -0.043 |
| SDQ12_5 ~~ | SDQ22_5 | 0.010 | 0.003 | 0.001 | 0.004 | 0.016 | 0.010 |
| SDQ18_5 ~~ | SDQ22_5 | 0.011 | 0.004 | 0.002 | 0.004 | 0.018 | 0.011 |
| SDQ7_4 ~~ | SDQ22_4 | -0.021 | 0.003 | <.001 | -0.027 | -0.014 | -0.021 |
| SDQ5_5 ~~ | SDQ22_5 | -0.007 | 0.005 | 0.17 | -0.017 | 0.003 | -0.007 |
| SDQ5_4 ~~ | SDQ18_4 | -0.032 | 0.006 | <.001 | -0.044 | -0.021 | -0.032 |
| ConductT3_lat ~~ | ConductT4_lat | 0.170 | 0.022 | <.001 | 0.127 | 0.214 | 0.170 |
| ConductT3_lat ~~ | ConductT5_lat | 0.133 | 0.025 | <.001 | 0.085 | 0.181 | 0.133 |
| ConductT4_lat ~~ | ConductT5_lat | 0.310 | 0.023 | <.001 | 0.264 | 0.356 | 0.310 |

**Table S4**

*Basic model: Testing Effect of Maternal and Partner-reported Intimate Partner Aggression on Child Conduct Problems – Estimates for Latent variables*

|  |  | Unstd. Estimate | Std. Error | *p*(>\|z\|) | CI lower | CI upper | Std. Estimate |
| --- | --- | --- | --- | --- | --- | --- | --- |
| ConductT3_lat |  |  |  |  |  |  |  |
|  | SDQ5_3 | 0.330 | 0.011 | <.001 | 0.308 | 0.352 | 0.338 |
|  | SDQ7_3 | 0.181 | 0.009 | <.001 | 0.163 | 0.200 | 0.185 |
|  | SDQ12_3 | 0.256 | 0.010 | <.001 | 0.237 | 0.275 | 0.262 |
|  | SDQ18_3 | 0.340 | 0.011 | <.001 | 0.319 | 0.361 | 0.347 |
|  | SDQ22_3 | 0.181 | 0.009 | <.001 | 0.163 | 0.200 | 0.185 |
| ConductT4_lat |  |  |  |  |  |  |  |
|  | SDQ5_4 | 0.376 | 0.014 | <.001 | 0.348 | 0.403 | 0.387 |
|  | SDQ7_4 | 0.200 | 0.010 | <.001 | 0.181 | 0.219 | 0.206 |
|  | SDQ12_4 | 0.228 | 0.008 | <.001 | 0.212 | 0.243 | 0.235 |
|  | SDQ18_4 | 0.263 | 0.010 | <.001 | 0.243 | 0.283 | 0.271 |
|  | SDQ22_4 | 0.185 | 0.010 | <.001 | 0.165 | 0.206 | 0.191 |
| ConductT5_lat |  |  |  |  |  |  |  |
|  | SDQ5_5 | 0.347 | 0.014 | <.001 | 0.320 | 0.375 | 0.354 |
|  | SDQ7_5 | 0.250 | 0.012 | <.001 | 0.227 | 0.273 | 0.255 |
|  | SDQ12_5 | 0.172 | 0.009 | <.001 | 0.155 | 0.189 | 0.175 |
|  | SDQ18_5 | 0.241 | 0.010 | <.001 | 0.221 | 0.261 | 0.246 |
|  | SDQ22_5 | 0.088 | 0.014 | <.001 | 0.062 | 0.115 | 0.090 |

**Table S5**

*Mediation Path model: Testing Mediating Effect of Maternal Depression on the Effect of Maternal and Partner-reported Intimate Partner Aggression on Child Conduct Problems - Covariances*

|  |  | Unstd. Estimate | Std. Error | *p*(>\|z\|) | CI lower | CI upper | Std. Estimate |
| --- | --- | --- | --- | --- | --- | --- | --- |
| IPA_MotherT1 ~~ | IPA_MotherT2 | 4.544 | 0.217 | <.001 | 4.119 | 4.969 | 4.544 |
| IPA_PartnerT1 ~~ | IPA_PartnerT2 | 3.447 | 0.147 | <.001 | 3.158 | 3.736 | 3.447 |
| IPA_MotherT1 ~~ | IPA_PartnerT1 | 3.204 | 0.167 | <.001 | 2.877 | 3.531 | 3.204 |
| IPA_MotherT2 ~~ | IPA_PartnerT2 | 4.233 | 0.212 | <.001 | 3.817 | 4.649 | 4.233 |
| IPA_MotherT1 ~~ | IPA_PartnerT2 | 3.113 | 0.175 | <.001 | 2.77 | 3.457 | 3.113 |
| IPA_MotherT2 ~~ | IPA_PartnerT1 | 2.964 | 0.138 | <.001 | 2.693 | 3.235 | 2.964 |
| SDQ5_3 ~~ | SDQ5_4 | 0.021 | 0.005 | <.001 | 0.011 | 0.031 | 0.021 |
| SDQ5_4 ~~ | SDQ5_5 | 0.065 | 0.005 | <.001 | 0.055 | 0.076 | 0.065 |
| SDQ5_3 ~~ | SDQ5_5 | 0.009 | 0.005 | 0.07 | -0.001 | 0.018 | 0.009 |
| SDQ7_3 ~~ | SDQ7_4 | 0.013 | 0.004 | <.001 | 0.006 | 0.02 | 0.013 |
| SDQ7_4 ~~ | SDQ7_5 | 0.033 | 0.004 | <.001 | 0.026 | 0.04 | 0.033 |
| SDQ7_3 ~~ | SDQ7_5 | 0.008 | 0.003 | 0.026 | 0.001 | 0.014 | 0.008 |
| SDQ12_3 ~~ | SDQ12_4 | <0.001 | 0.003 | 0.969 | -0.005 | 0.005 | <0.001 |
| SDQ12_4 ~~ | SDQ12_5 | -0.001 | 0.002 | 0.708 | -0.004 | 0.003 | -0.001 |
| SDQ12_3 ~~ | SDQ12_5 | 0.005 | 0.002 | 0.04 | <0.001 | 0.009 | 0.005 |
| SDQ18_3 ~~ | SDQ18_4 | -0.005 | 0.003 | 0.156 | -0.011 | 0.002 | -0.005 |
| SDQ18_4 ~~ | SDQ18_5 | 0.014 | 0.003 | <.001 | 0.008 | 0.02 | 0.014 |
| SDQ18_3 ~~ | SDQ18_5 | -0.005 | 0.003 | 0.125 | -0.011 | 0.001 | -0.005 |
| SDQ22_3 ~~ | SDQ22_4 | 0.001 | 0.002 | 0.745 | -0.004 | 0.005 | 0.001 |
| SDQ22_4 ~~ | SDQ22_5 | 0.004 | 0.002 | 0.047 | <0.001 | 0.007 | 0.004 |
| SDQ22_3 ~~ | SDQ22_5 | -0.001 | 0.002 | 0.477 | -0.004 | 0.002 | -0.001 |
| SDQ5_5 ~~ | SDQ7_5 | 0.024 | 0.006 | <.001 | 0.012 | 0.036 | 0.024 |
|  |  | Unstd. Estimate | Std. Error | *p*(>\|z\|) | CI lower | CI upper | Std. Estimate |
| SDQ12_3 ~~ | SDQ22_3 | 0.033 | 0.004 | <.001 | 0.025 | 0.041 | 0.033 |
| SDQ7_5 ~~ | SDQ22_5 | -0.006 | 0.004 | 0.075 | -0.013 | 0.001 | -0.006 |
| SDQ5_4 ~~ | SDQ22_4 | -0.042 | 0.005 | <.001 | -0.051 | -0.034 | -0.042 |
| SDQ12_5 ~~ | SDQ22_5 | 0.011 | 0.003 | <.001 | 0.006 | 0.016 | 0.011 |
| SDQ18_5 ~~ | SDQ22_5 | 0.013 | 0.003 | <.001 | 0.006 | 0.02 | 0.013 |
| SDQ7_4 ~~ | SDQ22_4 | -0.02 | 0.003 | <.001 | -0.027 | -0.014 | -0.02 |
| SDQ5_5 ~~ | SDQ22_5 | -0.005 | 0.005 | 0.302 | -0.014 | 0.004 | -0.005 |
| SDQ5_4 ~~ | SDQ18_4 | -0.033 | 0.006 | <.001 | -0.044 | -0.021 | -0.033 |
| ConductT3_lat ~~ | ConductT4_lat | 0.170 | 0.022 | <.001 | 0.127 | 0.214 | 0.170 |
|  | ConductT5_lat | 0.130 | 0.025 | <.001 | 0.081 | 0.178 | 0.130 |
| ConductT4_lat ~~ | ConductT5_lat | 0.299 | 0.024 | <.001 | 0.252 | 0.346 | 0.299 |

**Table S6**

*Mediation Path model: Testing Mediating Effect of Maternal Depression on the Effect of Maternal and Partner-reported Intimate Partner Aggression on Child Conduct Problems – Estimates for Latent variables*

|  |  | Unstd. Estimate | Std. Error | *p*(>\|z\|) | CI lower | CI upper | Std. Estimate |
| --- | --- | --- | --- | --- | --- | --- | --- |
| ConductT3_lat |  |  |  |  |  |  |  |
|  | SDQ5_3 | 0.328 | 0.011 | <.001 | 0.306 | 0.350 | 0.336 |
|  | SDQ7_3 | 0.179 | 0.009 | <.001 | 0.160 | 0.197 | 0.183 |
|  | SDQ12_3 | 0.257 | 0.009 | <.001 | 0.238 | 0.275 | 0.262 |
|  | SDQ18_3 | 0.338 | 0.011 | <.001 | 0.317 | 0.358 | 0.345 |
|  | SDQ22_3 | 0.182 | 0.009 | <.001 | 0.164 | 0.201 | 0.186 |
| ConductT4_lat |  |  |  |  |  |  |  |
|  | SDQ5_4 | 0.376 | 0.014 | <.001 | 0.348 | 0.403 | 0.387 |
|  | SDQ7_4 | 0.200 | 0.010 | <.001 | 0.181 | 0.219 | 0.206 |
|  | SDQ12_4 | 0.228 | 0.008 | <.001 | 0.212 | 0.243 | 0.235 |
|  | SDQ18_4 | 0.263 | 0.010 | <.001 | 0.243 | 0.283 | 0.271 |
|  | SDQ22_4 | 0.185 | 0.010 | <.001 | 0.165 | 0.206 | 0.191 |
| ConductT5_lat |  |  |  |  |  |  |  |
|  | SDQ5_5 | 0.347 | 0.014 | <.001 | 0.320 | 0.375 | 0.354 |
|  | SDQ7_5 | 0.250 | 0.012 | <.001 | 0.227 | 0.273 | 0.255 |
|  | SDQ12_5 | 0.172 | 0.009 | <.001 | 0.155 | 0.189 | 0.175 |
|  | SDQ18_5 | 0.241 | 0.010 | <.001 | 0.221 | 0.261 | 0.246 |
|  | SDQ22_5 | 0.088 | 0.014 | <.001 | 0.062 | 0.115 | 0.090 |

**Table S7**

*Estimates for Direct, Indirect and Total Effects for the Moderating Role of Maternal Warmth for the Mediation by Maternal Depression for the Effect of Maternal- and Partner-Reported Intimate Partner Aggression on Child Conduct Problems*

| Regression paths |  | | Unstd. Estimate | | Std. Error | | *p* | | CI lower | | CI upper | | Std. Estimate | |
| --- | --- | --- | --- | --- | --- | --- | --- | --- | --- | --- | --- | --- | --- | --- |
| IPA M T1 | Conduct T3 | | -0.002 | | 0.009 | | .807 | | -0.021 | | 0.016 | | -0.002 | |
|  | Conduct T4 | | 0.039 | | 0.009 | | <.001 | | 0.020 | | 0.057 | | 0.038 | |
|  | Conduct T5 | | 0.017 | | 0.011 | | .117 | | -0.004 | | 0.038 | | 0.017 | |
| IPA M T2 | Conduct T3 | | -0.006 | | 0.009 | | .485 | | -0.024 | | 0.011 | | -0.006 | |
|  | Conduct T4 | | 0.051 | | 0.009 | | <.001 | | 0.034 | | 0.069 | | 0.050 | |
|  | Conduct T5 | | 0.048 | | 0.009 | | <.001 | | 0.030 | | 0.067 | | 0.048 | |
| IPA P T1 | Conduct T3 | | 0.027 | | 0.010 | | .007 | | 0.007 | | 0.048 | | 0.027 | |
|  | Conduct T4 | | -0.007 | | 0.011 | | .493 | | -0.029 | | 0.014 | | -0.007 | |
|  | Conduct T5 | | -0.010 | | 0.011 | | .378 | | -0.032 | | 0.012 | | -0.010 | |
| IPA P T2 | Conduct T3 | | 0.064 | | 0.010 | | <.001 | | 0.045 | | 0.084 | | 0.063 | |
|  | Conduct T4 | | 0.024 | | 0.010 | | .019 | | 0.004 | | 0.043 | | 0.023 | |
|  | Conduct T5 | | 0.007 | | 0.011 | | .482 | | -0.013 | | 0.028 | | 0.007 | |
| Indirect effects | | | | | | | | | | | | | | |
| IPA M T1- Mat Dep T4 - Conduct T5 | | | 0.003 | | 0.001 | | .010 | | 0.001 | | 0.004 | | 0.002 | |
| IPA M T2- Mat Dep T4 - Conduct T5 | | | 0.003 | | 0.001 | | .006 | | 0.001 | | 0.005 | | 0.003 | |
| Regression paths |  | | Unstd. Estimate | | Std. Error | | *p* | | CI lower | | CI upper | | Std. Estimate | |
| Total effects | | | | | | | | | | | | | | |
| IPA M T1- Mat Dep T4 - Conduct T5 | | 0.019 | | 0.011 | | .070 | | -0.002 | | 0.040 | | 0.019 | |  |
| IPA M T2- Mat Dep T4 - Conduct T5 | | 0.051 | | 0.009 | | <.001 | | 0.033 | | 0.070 | | 0.051 | |  |
| IPA M T1- Mat Dep T4 - *Mat Warmth T4 * Mat Dep T4 | | -0.001 | | 0.002 | | .413 | | -0.005 | | 0.002 | | -0.001 | |  |
| IPA M T2- Mat Dep T4 - *Mat Warmth T4 * Mat Dep T4 | | -0.002 | | 0.002 | | .416 | | -0.006 | | 0.002 | | -.002 | |  |

*Note: T1 = antenatal, T2 = 9 months, T3 = 2 years, T4 = 4.5 years, T5 = 8 years, IPA-M = Mother-reported Intimate Partner Aggression, IPA P = Partner reported Intimate Partner Aggression, Std. Estimates are level standardized.*

**Figure S1**

*Moderated Mediation Path model: Testing Moderating Effect of Maternal Warmth on the Mediating Effect of Maternal Depression on the Effect of Maternal and Partner-reported Intimate Partner Aggression on Child Conduct Problems - Covariances*


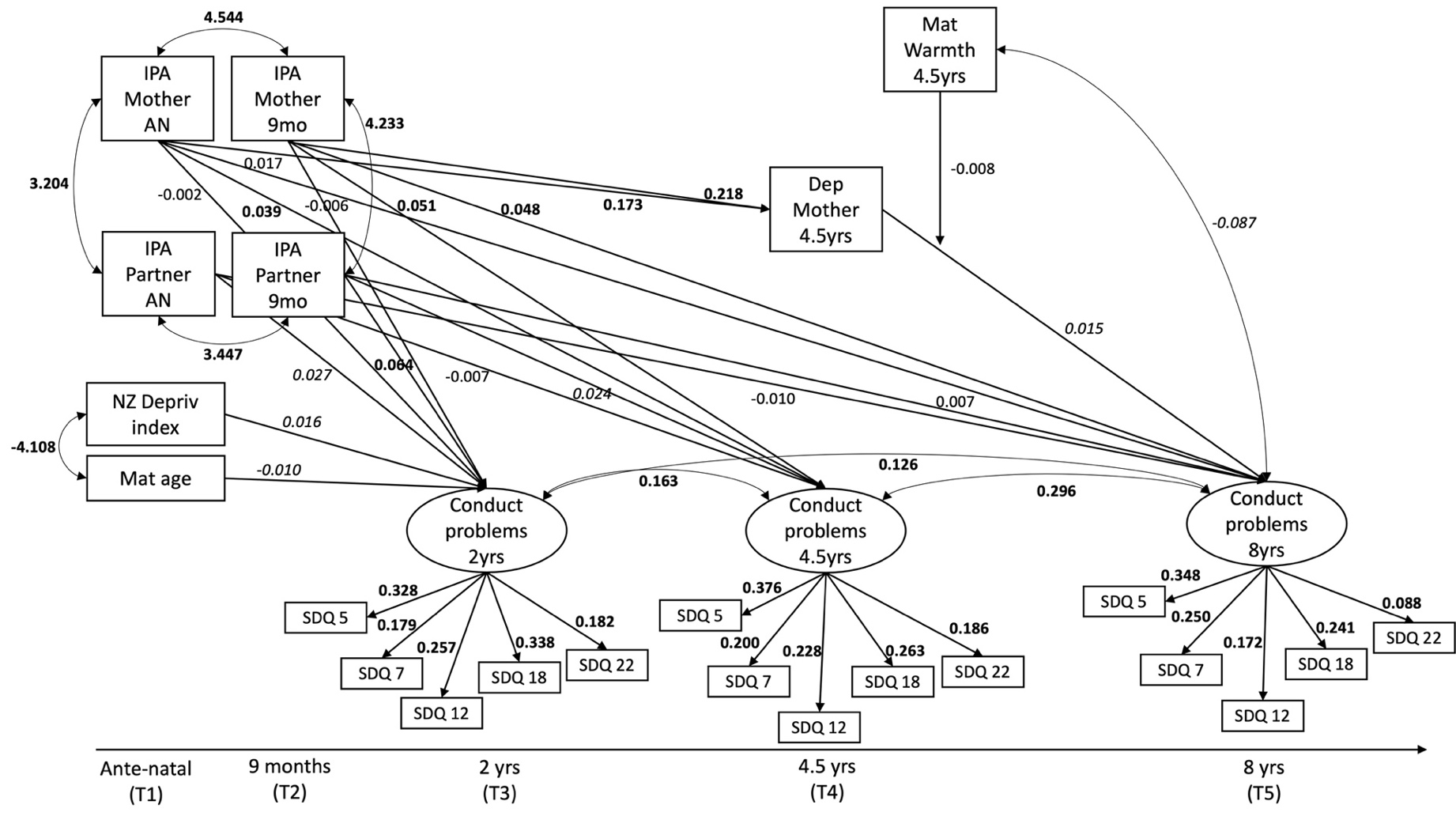


*Note: IPA = Intimate Partner Aggression, SDQ = Strengths and Difficulties Questionnaire, covariances between each item’s timepoints are not shown for readability, estimates at p <.001, except when italicized, then p <.05.*
